# Supplementary material for: A Highly Conserved Shh Enhancer Coordinates Hypothalamic and Craniofacial Development
Source: Front Cell Dev Biol. 2021 Apr 1;9:595744. doi: 10.3389/fcell.2021.595744 (PMC8047142; doi:10.3389/fcell.2021.595744)
Supplement: Supplementary file 1 [file Data_Sheet_1.docx]

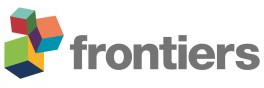


***Supplementary Material***


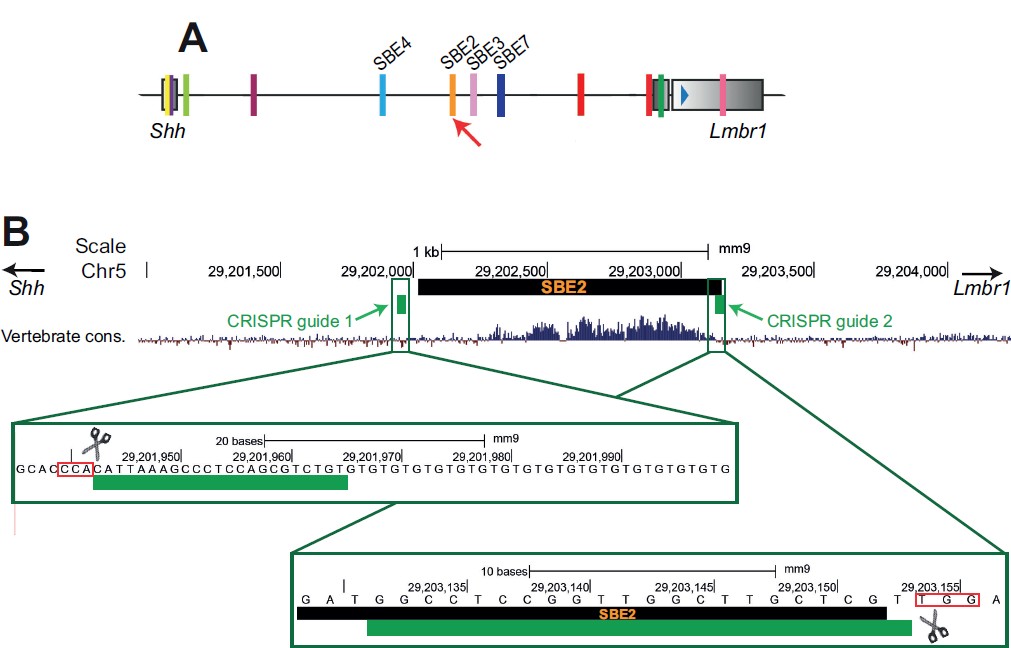


**Supplementary Figure 1. Overview of the region targeted via CRISPR for removal of SBE2 activity.** (A) Depiction of the *Shh* regulatory region. Coloured blocks represent the known *Shh* enhancers with the relevant brain enhancers labelled. The red arrow points to the location of the targeted SBE2 enhancer. (B) UCSC publicly available track of the vertebrate conservation for SBE2 and the neighbouring genomic region ([https://genome.ucsc.edu/cgi-](https://genome.ucsc.edu/cgi-bin/hgTrackUi?db=hg19&g=cons100way) [bin/hgTrackUi?db=hg19&g=cons100way](https://genome.ucsc.edu/cgi-bin/hgTrackUi?db=hg19&g=cons100way)). CRISPR guides are represented by the green blocks. A zoomed in view of each CRISPR guide and the targeted sequence is presented in the green boxes. The pam sites are demarcated by the red boxes.


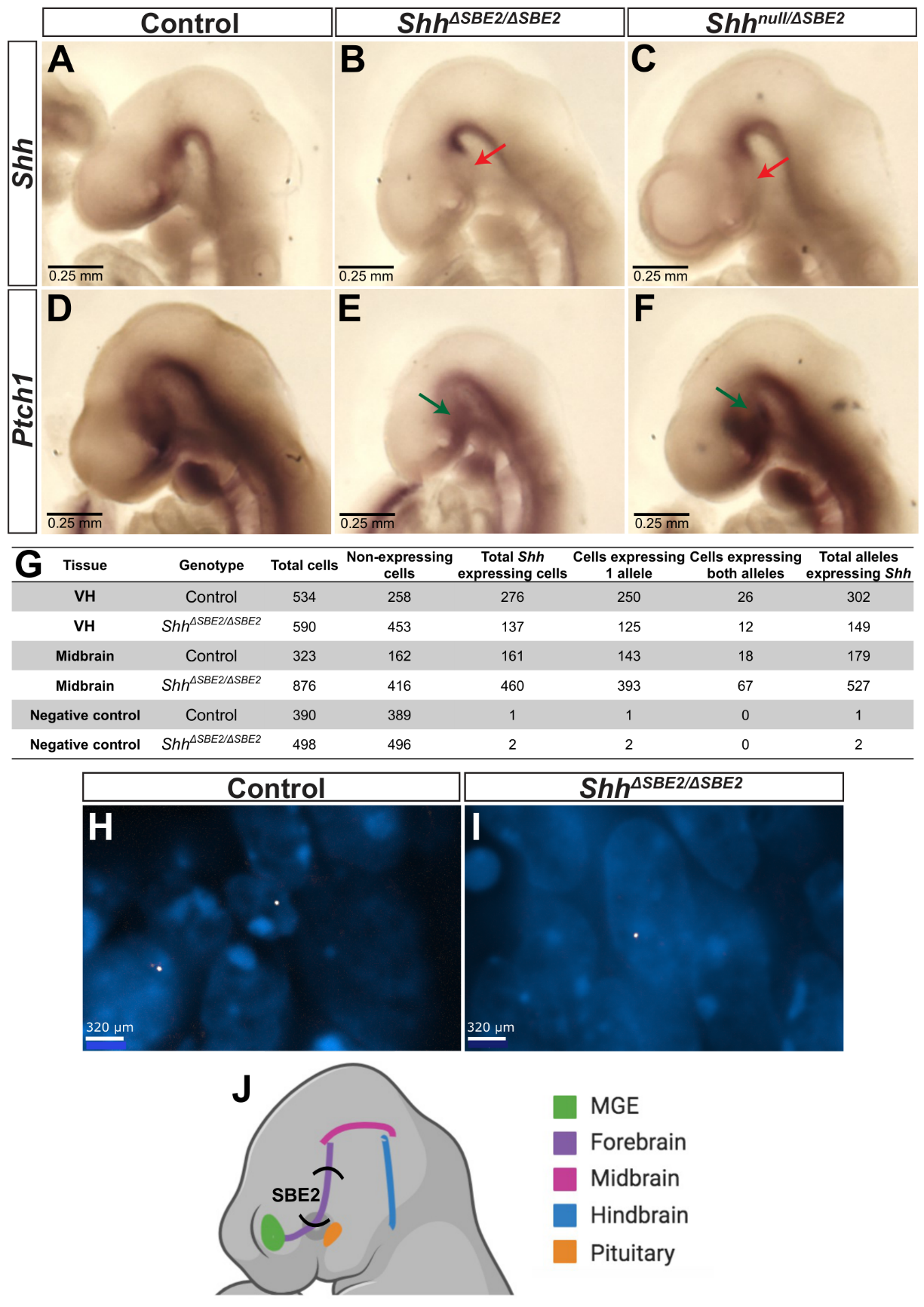


**Supplementary Figure 2. Decreased *Shh* activity in the rostral diencephalon upon removal of SBE2.** (A-C) *In situ* hybridisation for *Shh* in E9.5 embryos is depicted for control, *Shh^ΔSBE2/ΔSBE2^* homozygotes and *Shh^null/ΔSBE2^* compound heterozygotes. The red arrows point to the rostral diencephalic region where *Shh* expression is lost. (D-F) *In situ* hybridisation for *Ptch1* in E9.5 embryos is shown. The green arrows point to the rostral diencephalon. Scale bars (A-F) appear in the bottom left hand corner. (G) Cell counts for FISH signal for the *Shh* allele in the ventral hypothalamus (VH), midbrain and negative control tissue at E10.5. (H-I) RNA FISH signal detected in control (H) and *Shh^null/ΔSBE2^* mutant (I) VH tissue at E10.5. Scale bars (H-I) appear in the bottom left hand corner. (J) Schematic diagram depicting the region of *Shh* expression affected upon removal of SBE2. (n=3)


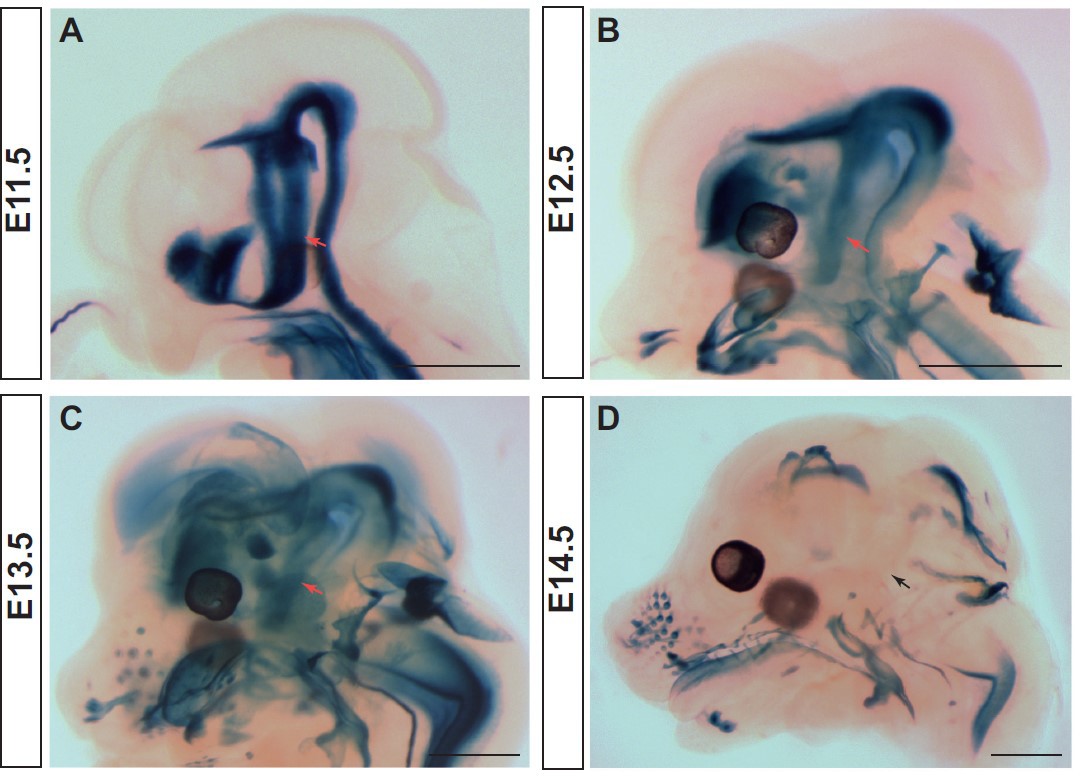
**Supplementary Figure 3. *Shh* expression is present within the VH up to E13.5.** (A-D) *lacZ* reporter gene expression in E11.5 (A), E12.5 (B), E13.5 (C) and E14.5 (D) BABB cleared embryonic heads of SBLac526 embryos representing regions of *Shh* expression. Red arrows point to VH expression, while the black arrow points to the VH region where no expression is seen at E14.5. (n=3)


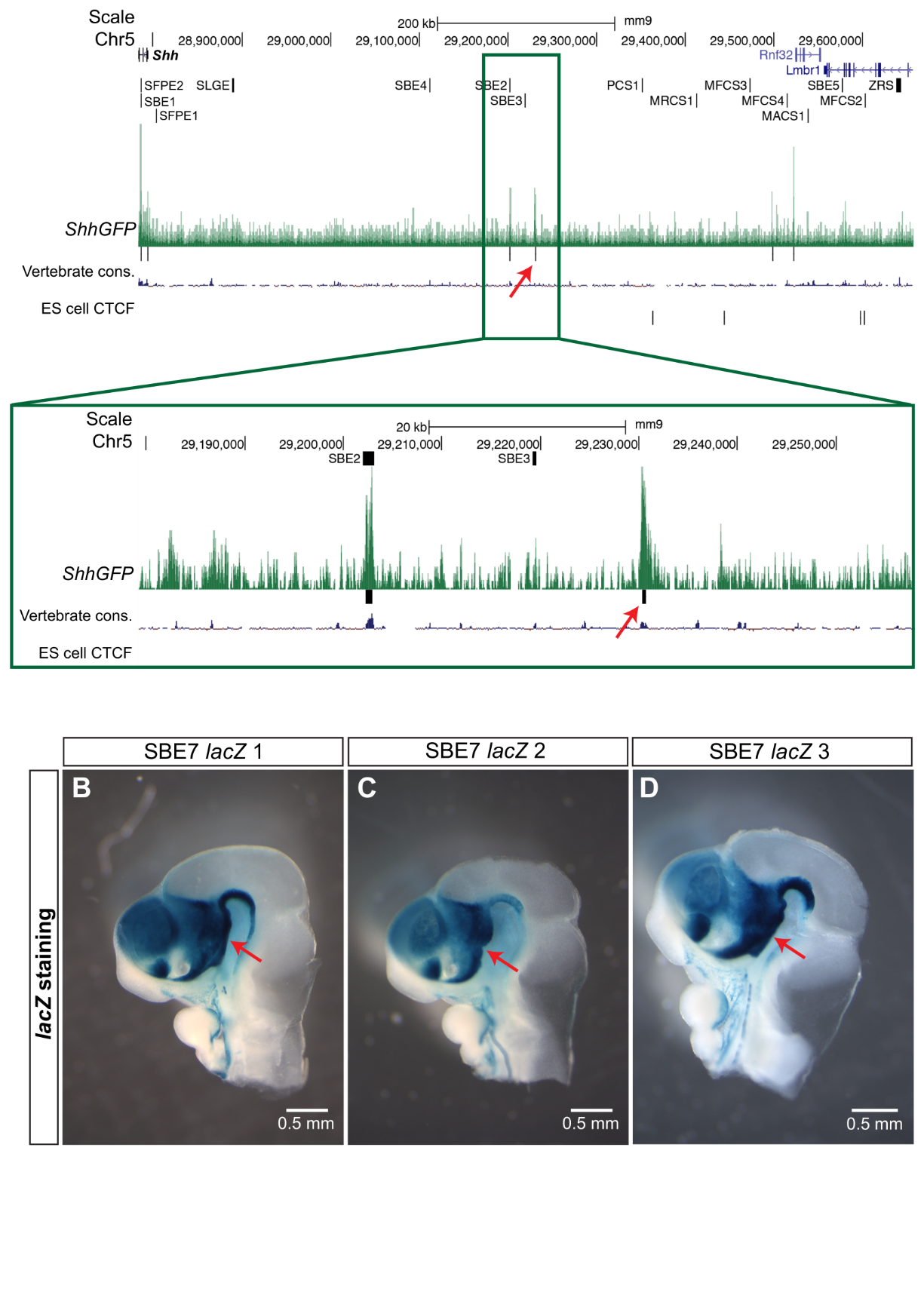


**Supplementary Figure 4. SBE7 is likely to drive residual *Shh* expression seen in the rostral diencephalon upon removal of SBE2 activity.** (A) UCSC track of ATAC-seq data from GFP positive cells isolated from the entire head of E10.5 *ShhGFP* embryos. The publicly available vertebrate conservation and CTCF signal from sorted from BRUCE-4 ES cell peak tracks are also presented as are the sites for the known *Shh* conserved enhancers. The red arrows point to the peak detected in *Shh* expressing cells of the brain identified as SBE7. A zoomed in view of the detected peak is presented in the green box depicting the conservation seen in this region more closely. (B-D) *lacZ* expression in E10.5 day heads (bisected along the midline) from three transgenics is shown, where *lacZ* expression is under the control of SBE7 from (A). Red arrows (in B-D) point to the region of the rostral diencephalon where *lacZ* expression is detected. Scale bars are shown in the bottom right hand corner.


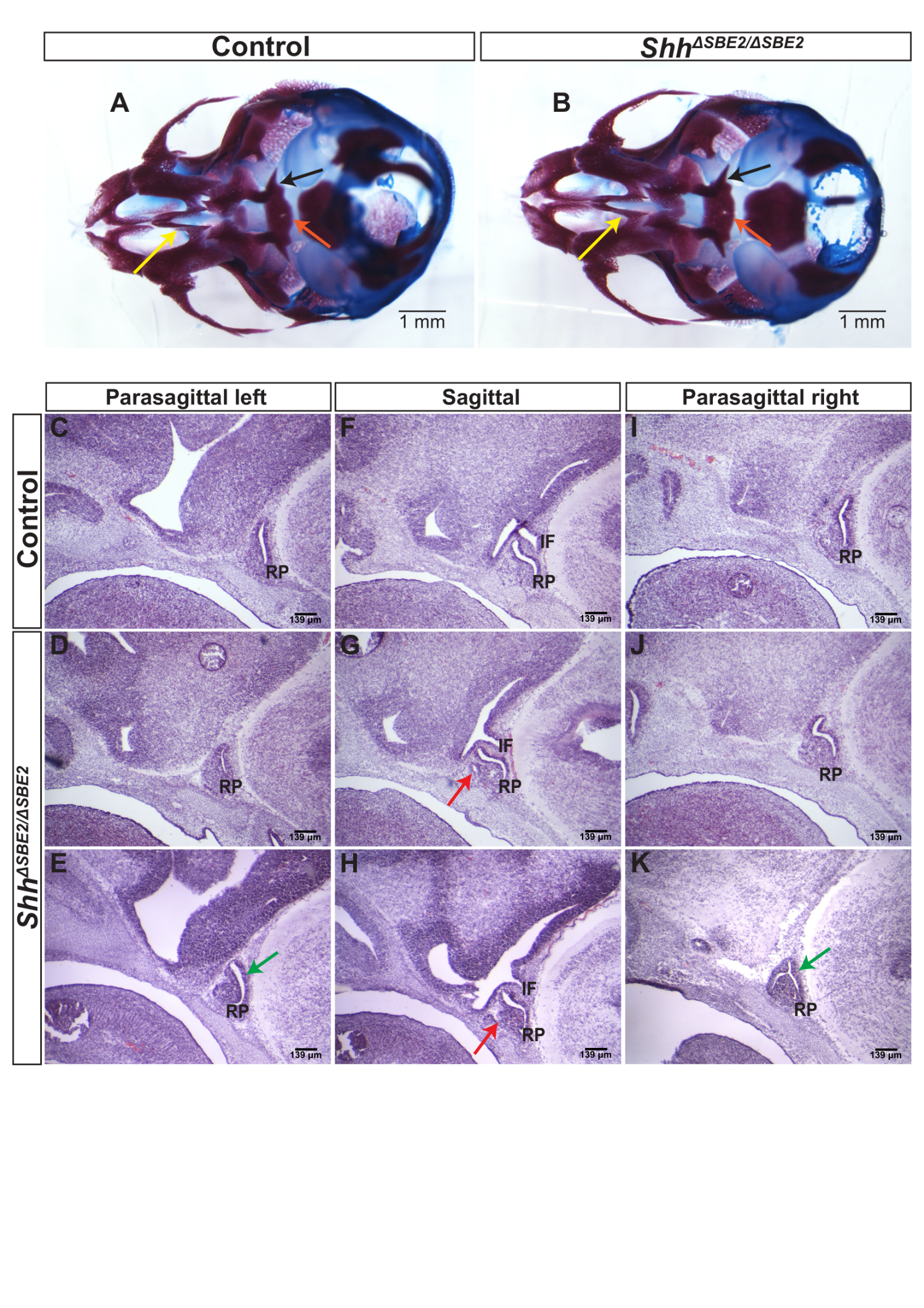


**Supplementary Figure 5. *ShhΔSBE2/ΔSBE2* embryos lack malformations of the bones of the cranial vault and present mild lateral pituitary deformations.** (A-B) Dual staining highlighting chondrogenic (blue) and skeletal (red) craniofacial elements of control and *ShhΔSBE2/ΔSBE2* E17.5 heads are shown. Orange arrows point to the basisphenoid bone, yellow arrows indicate the vomer bone and the black arrows point to the pterygoid bone. (C-K) H&E staining of E13.5 day left and right parasagittal and sagittal cryosections from control and two *ShhΔSBE2/ΔSBE2* heads. Green arrows point to the mild lateral adenohypophyseal malformations seen in some *ShhΔSBE2/ΔSBE2* mutant embryos. Red arrows point to the subtle midline adenohypophyseal malformations seen in *ShhΔSBE2/ΔSBE2* embryos. RP- Rathke’s pouch; IF- infundibulum. Scale bars are shown in the bottom right hand corner. (n=3)


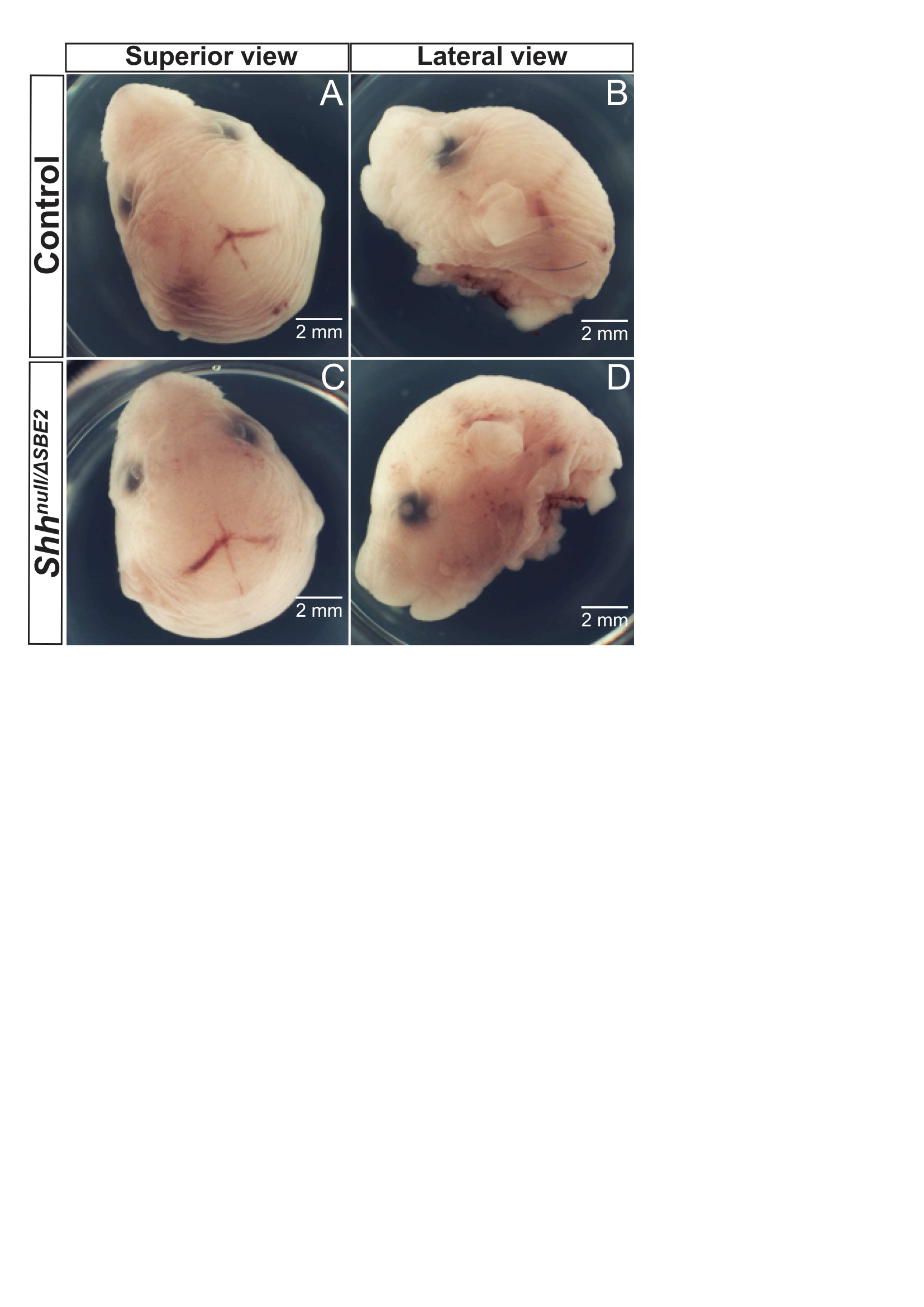


**Supplementary Figure 6. *Shhnull/ΔSBE2* embryos present subtle changes to overall head shape.** (A-B) External superior (A) and lateral (B) view of E17.5 control heads. (C-D) External superior (C) and lateral (D) view of E17.5 *Shhnull/ΔSBE2* heads. Scale bars are shown in the bottom right hand corner. (n=3)


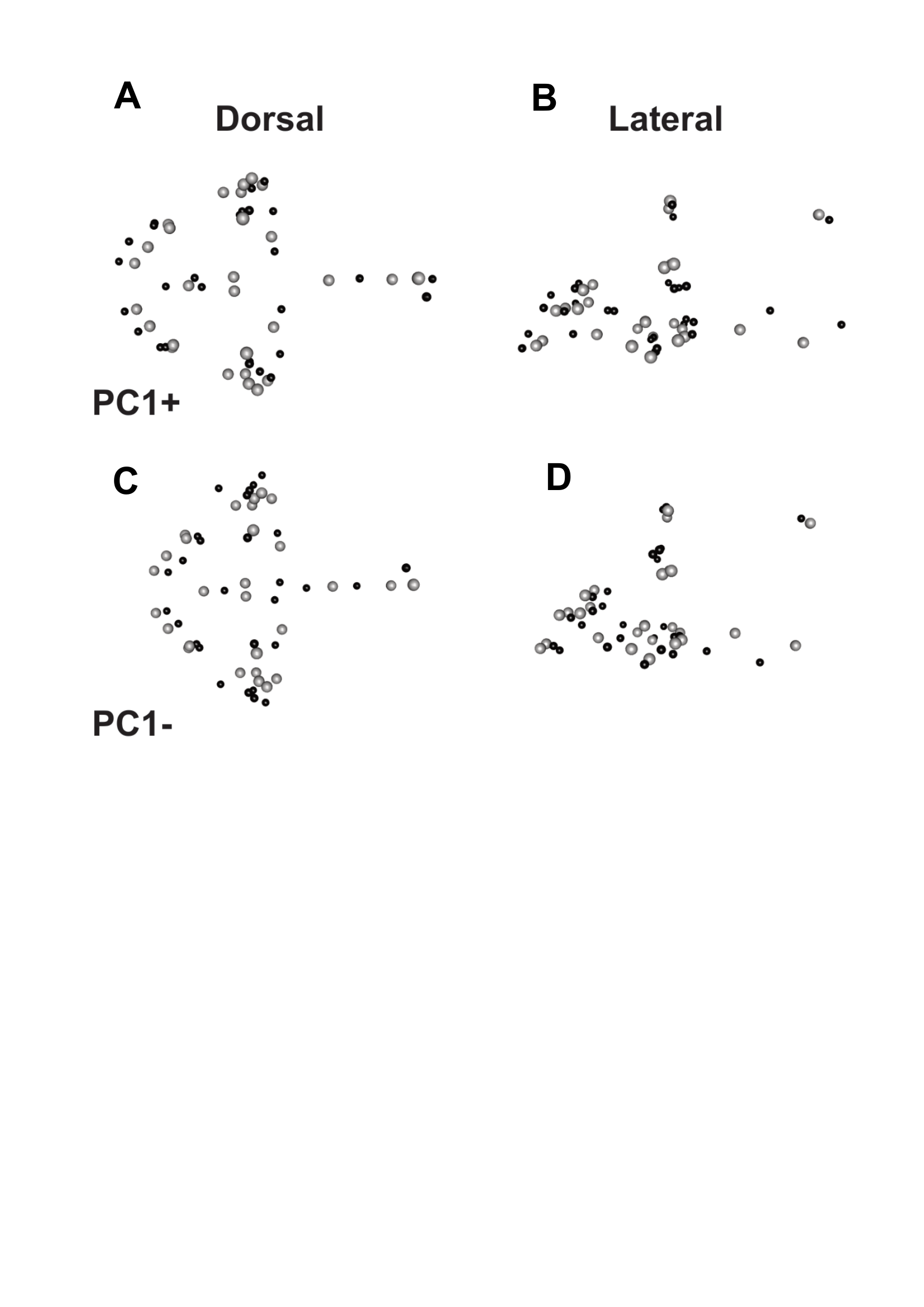


**Supplementary Figure 7. Landmark points used in the morphometric analysis.** Point plots illustrate the mean landmark configuration (black spheres) relative to the landmark configuration of extreme positive or negative PC1 (grey spheres), for the dorsal view with (A) and lateral view (B) and these same points when morphed for PC1 presented for the dorsal (C) and lateral (D) view. (control n=6; mutant n=8)


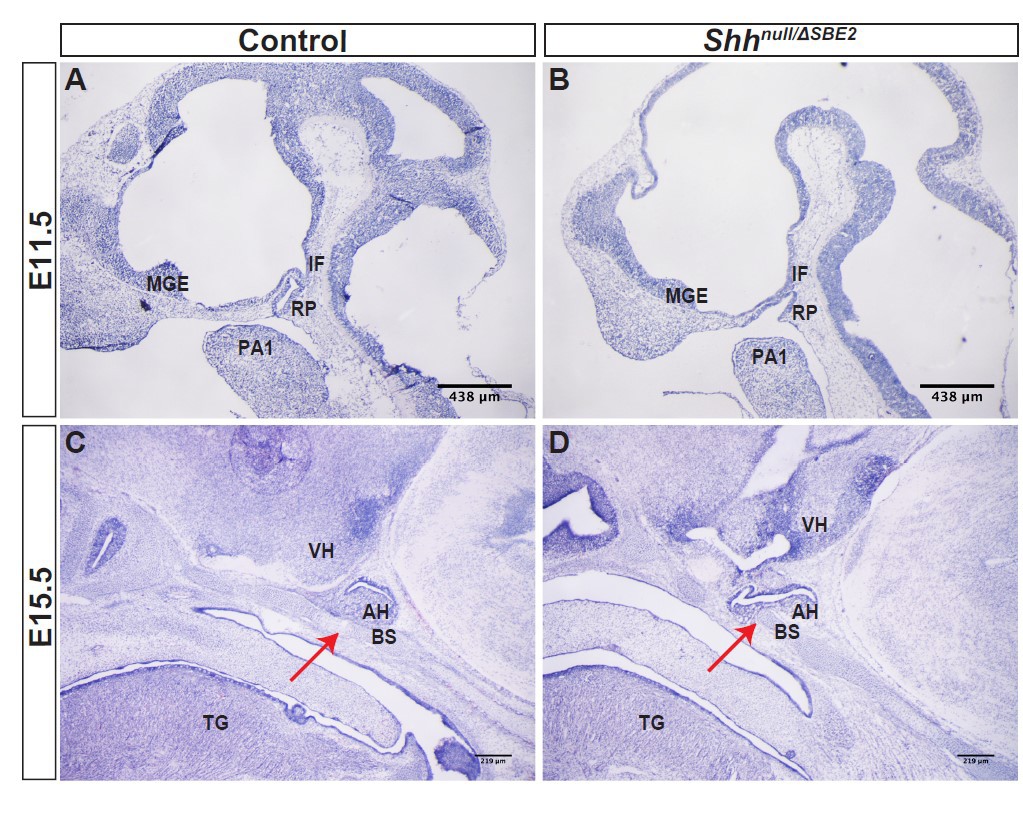


**Supplementary Figure 8. *Shhnull/ΔSBE2* embryos present ventrally shifted pituitary lobes which impede basisphenoid bone formation.** (A-B) H&E stained control and *Shhnull/ΔSBE2* E11.5 head cryosections are shown. (C-D) H&E stained control and *Shhnull/ΔSBE2* E15.5 heads cryosections are depicted. Red arrows point to the region of the basisphenoid bone intercepted by the adenohypophysis in mutant embryos. RP- Rathke’s pouch; IF- infundibulum; MGE- medial ganglionic eminence; PA1- pharyngeal arch 1; VH- ventral hypothalamus; BS- basisphenoid bone; TG- tongue; AH- adenohypophysis. Scale bars are shown in the bottom right hand corner. (n=3)


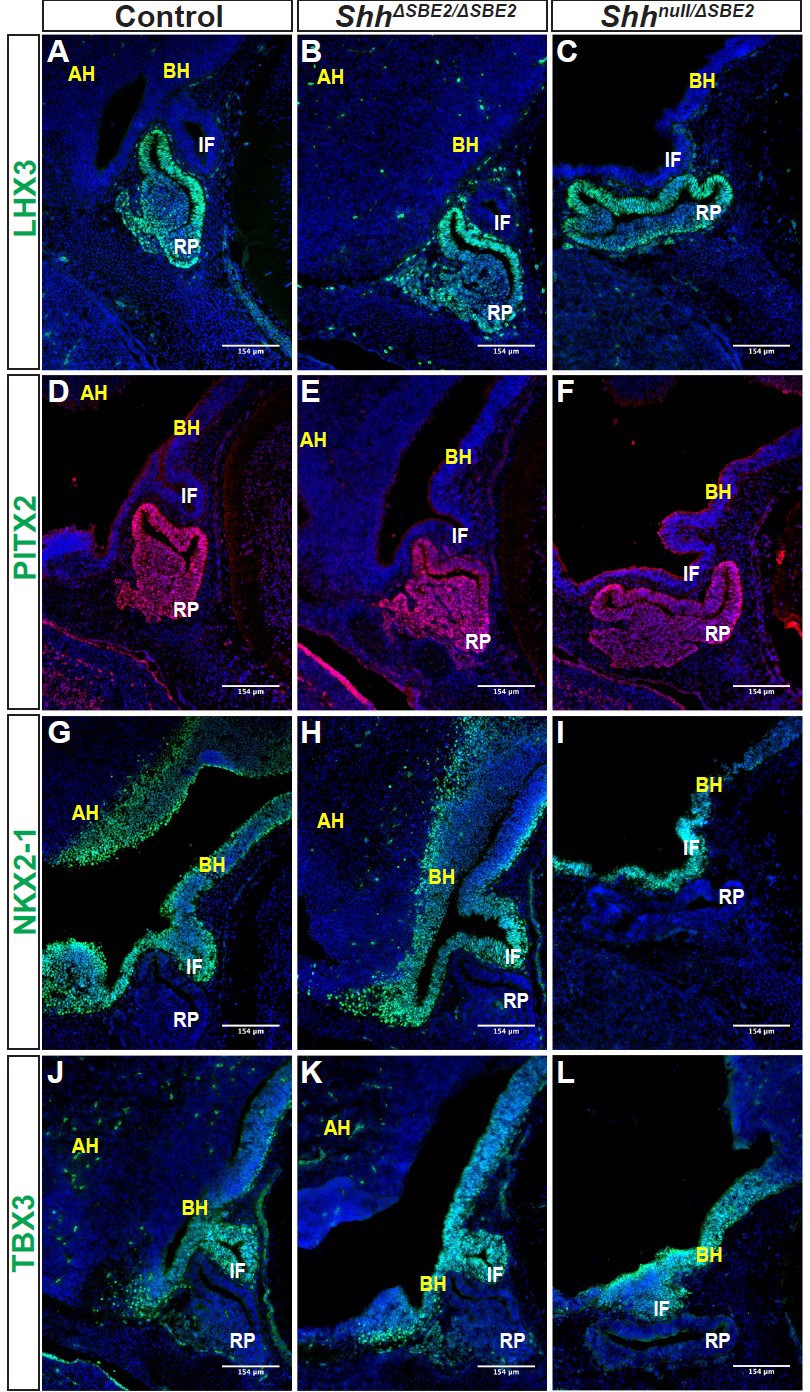


**Supplementary Figure 9. Patterning within the pituitary lobes is unaffected by loss of SBE2.** (A-C) Immunofluorescent staining for LHX3 in E13.5 day hypophyseal parasagittal cryosections of control, *ShhΔSBE2/ΔSBE2* and *Shhnull/ΔSBE2* embryos. (D-F) Immunofluorescent staining for PITX2 in E13.5 day hypophyseal parasagittal cryosections of control, *ShhΔSBE2/ΔSBE2* and *Shhnull/ΔSBE2* embryos. (G-I) Immunofluorescent staining for NKX2-1 in E13.5 day hypophyseal parasagittal cryosections of control, *ShhΔSBE2/ΔSBE2* and *Shhnull/ΔSBE2* embryos. (J-L) Immunofluorescent staining for TBX3 in E13.5 day hypophyseal parasagittal cryosections of control, *ShhΔSBE2/ΔSBE2* and *Shhnull/ΔSBE2* embryos. AH- alar hypothalamus; BH- basal hypothalamus; IF- infundibulum; RP- Rathke’s pouch. Scale bars are shown in the bottom right hand corner. (n=3)


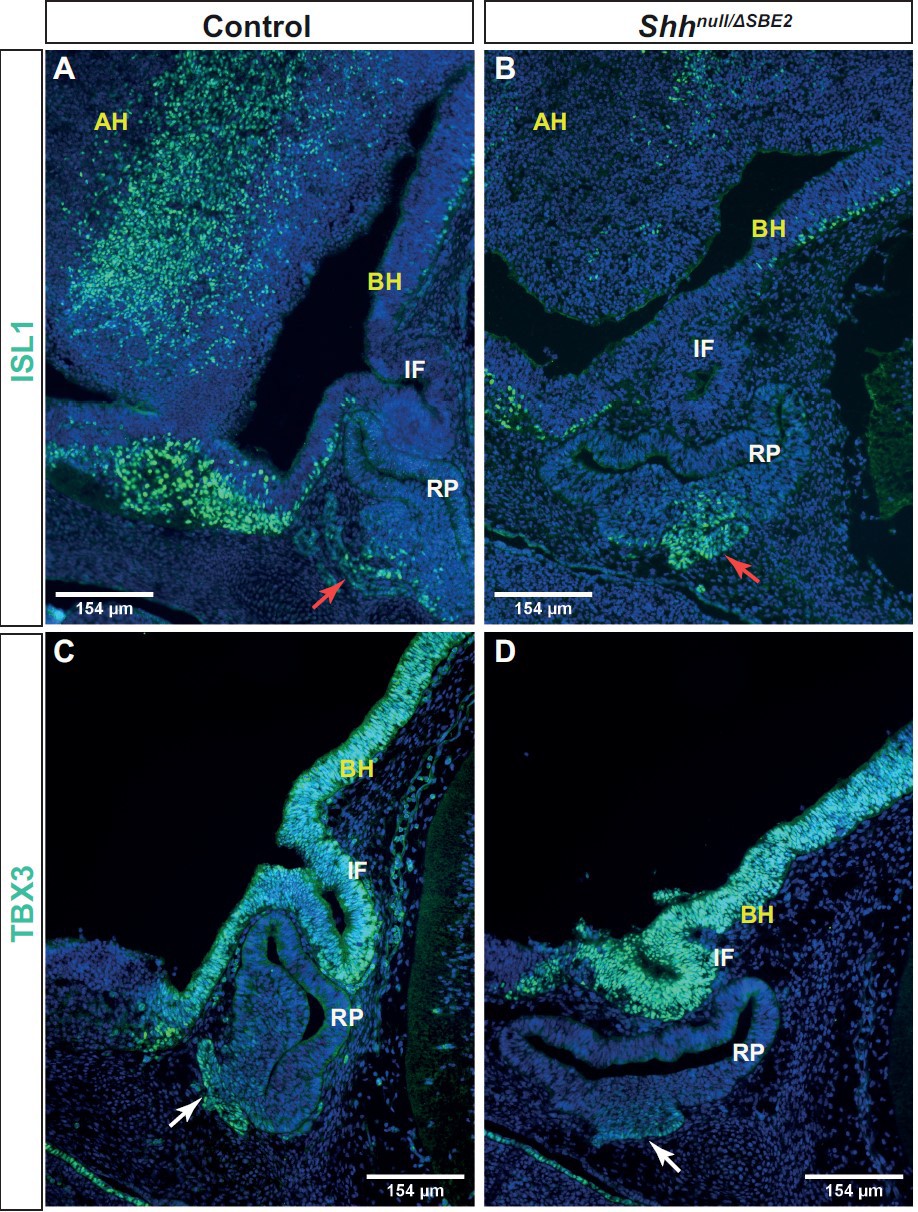


**Supplementary Figure 10. Loss of SBE2 does not lead to developmental delay of the adenohypophysis.** (A-B) Immunofluorescent staining for ISL1 in E12.5 day hypophyseal sagittal cryosections of control and *Shhnull/ΔSBE2* embryos. Red arrows point to the positive cell population in the ventral most portion of the adenohypophysis. (C-D) Immunofluorescent staining for TBX3 in E12.5 day hypophyseal parasagittal cryosections of control and *Shhnull/ΔSBE2* embryos. White arrows point to the positive staining in the ventral region of the adenohypophysis. BH- basal hypothalamus; IF- infundibulum; RP- Rathke’s pouch. Scale bars are shown in the bottom left/right hand corner. (n=3)


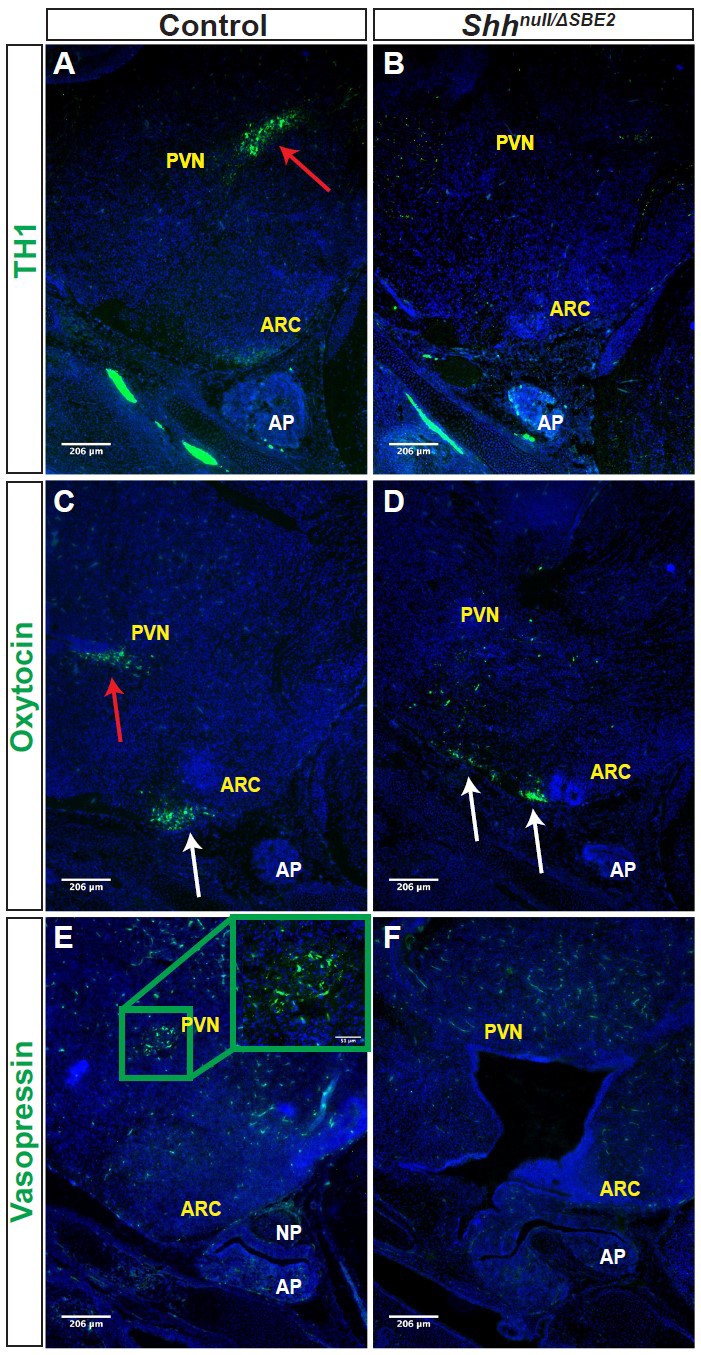


**Supplementary Figure 11. Neuronal populations which communicate with the hypophyseal lobes are affected in *Shhnull/ΔSBE2* embryos.** (A-B) Immunofluorescent staining for TH1 in E17.5 day VH parasagittal cryosections of control and *Shhnull/ΔSBE2* embryos. Red arrows point to the PVN TH1 neuronal population which is absent in *Shhnull/ΔSBE2* embryos. (C-D) Immunofluorescent staining for oxytocin in E17.5 day VH parasagittal cryosections of control and *Shhnull/ΔSBE2* embryos. Red arrows point to the PVN oxytocin neuronal population which is lost in *Shhnull/ΔSBE2* embryos. White arrows point to the ARC oxytocin neuronal population which is mislocalised in *Shhnull/ΔSBE2* embryos. (E-F) Immunofluorescent staining for vasopressin in E17.5 day VH parasagittal cryosections of control and *Shhnull/ΔSBE2* embryos. The green box presents the region of PVN expression of vasopressin which is absent in mutant embryos. A zoomed in view of the region of expression presented is seen in the upper right hand corner. NP- neurohypophysis; AP- adenohypophysis; PVN- paraventricular nucleus; ARC- arcuate nucleus. Scale bars are shown in the bottom left hand corner. (n=3)


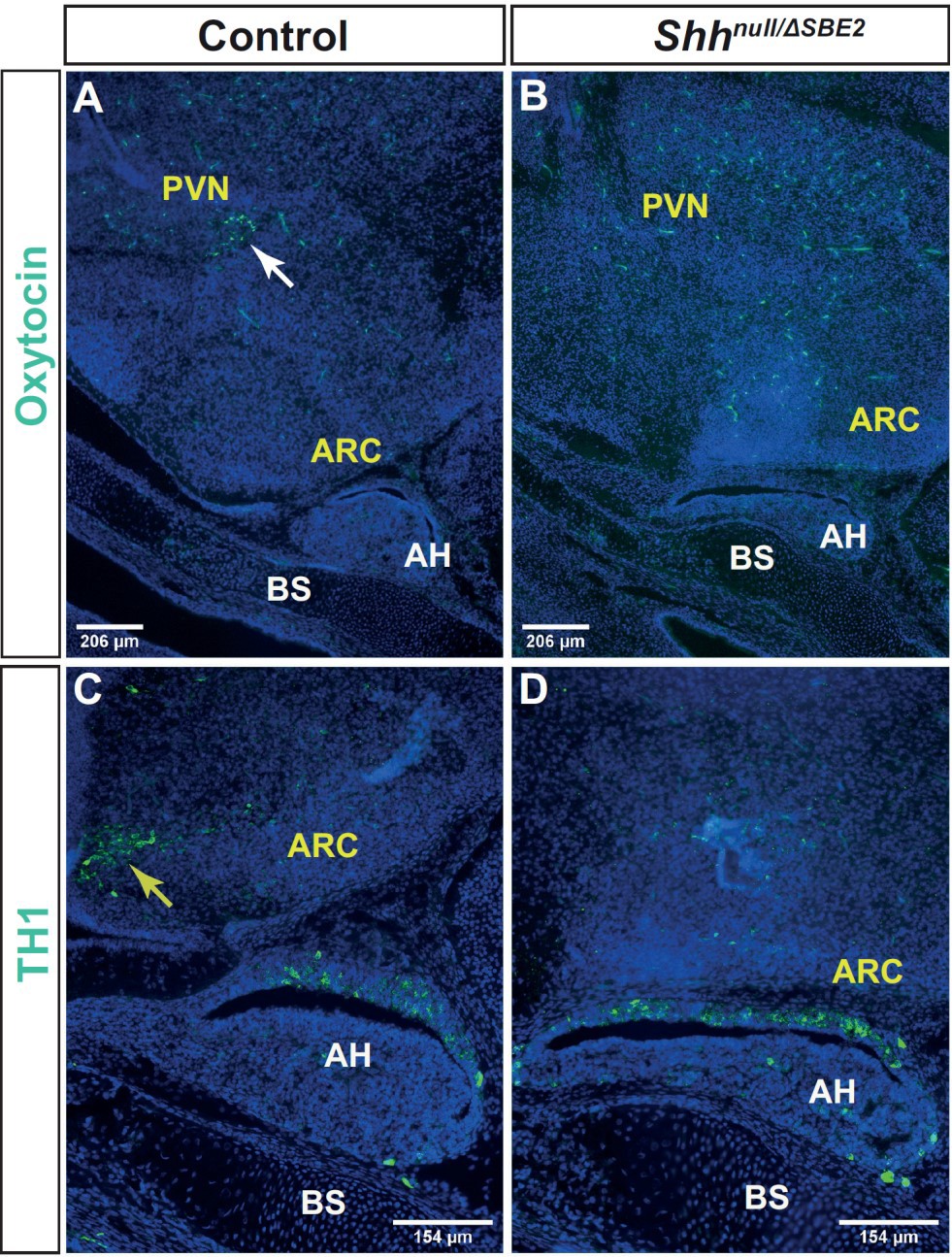


**Supplementary Figure 12. Loss of SBE2 does not cause a delay in hypothalamic neuronal specification.** (A-B) Immunofluorescent staining for oxytocin in E15.5 day VH parasagittal cryosections of control and *Shhnull/ΔSBE2* embryos. The white arrow points to the oxytocin population, lost in mutant embryos. (C-D) Immunofluorescent staining for TH1 in E15.5 day VH parasagittal cryosections of control and *Shhnull/ΔSBE2* embryos. The yellow arrow indicates the TH1 population, absent in mutant embryos. BS- basisphenoid; AH- adenohypophysis; PVN- paraventricular nucleus; ARC- arcuate nucleus. Scale bars are shown in the bottom left hand corner. (n=3)


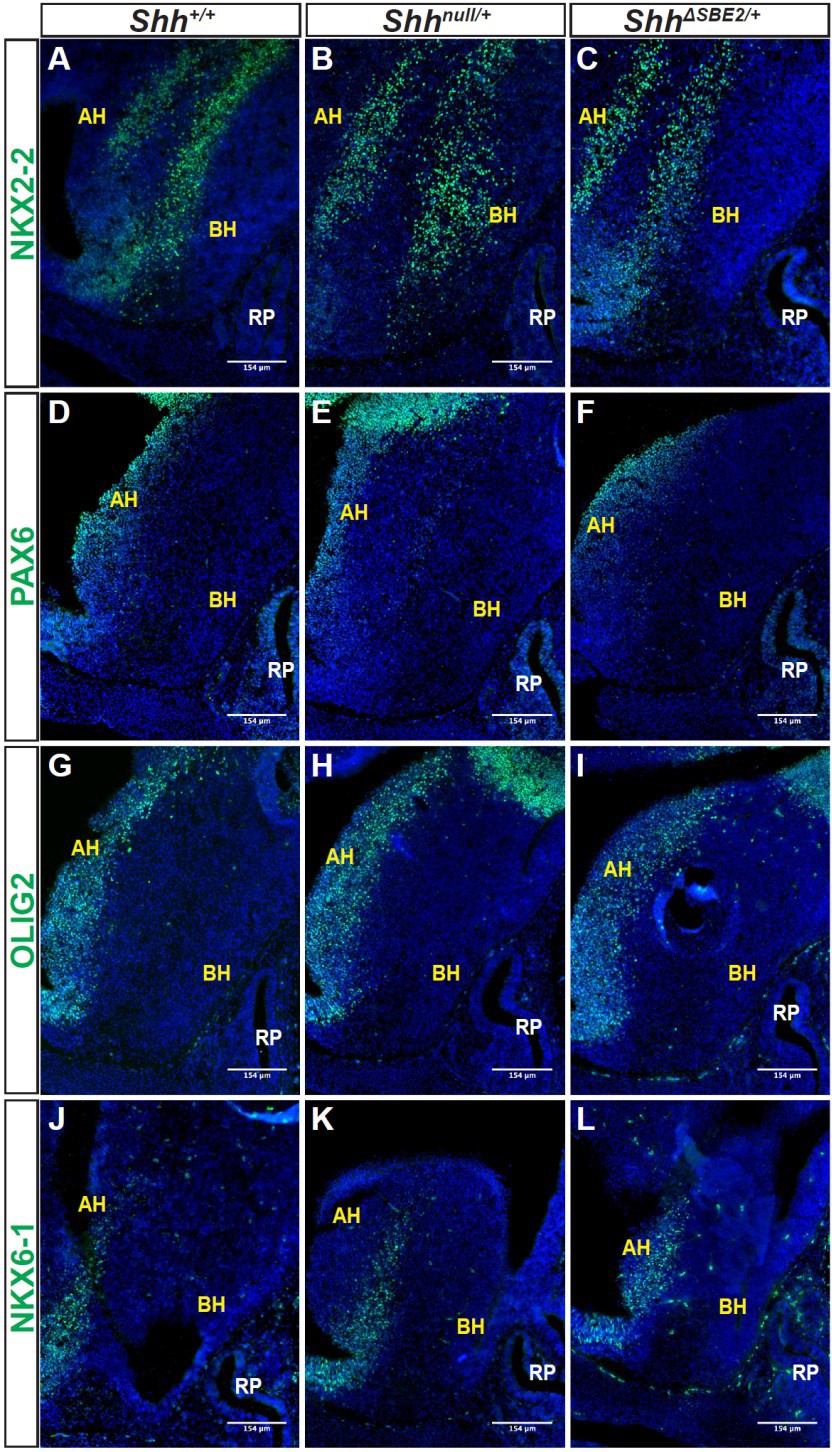


**Supplementary Figure 13. DV hypothalamic patterning is unaffected by heterozygous removal of SBE2 or *Shh* activity.** (A-C) Immunofluorescent staining for NKX2-2 in E13.5 day VH parasagittal cryosections of *Shh+/+*, *Shhnull/+* and *ShhΔSBE2/+* embryos. (D-F) Immunofluorescent staining for PAX6 in E13.5 day VH parasagittal cryosections of *Shh+/+*, *Shhnull/+* and *ShhΔSBE2/+* embryos. (G-I) Immunofluorescent staining for OLIG2 in E13.5 day VH parasagittal cryosections of *Shh+/+*, *Shhnull/+* and *ShhΔSBE2/+* embryos. (J-L) Immunofluorescent staining for NKX6-1 in E13.5 day VH parasagittal cryosections of *Shh+/+*, *Shhnull/+* and *ShhΔSBE2/+* embryos. AH- alar hypothalamus; BH- basal hypothalamus; RP- Rathke’s pouch. Scale bars are shown in the bottom right hand corner. (n=3)


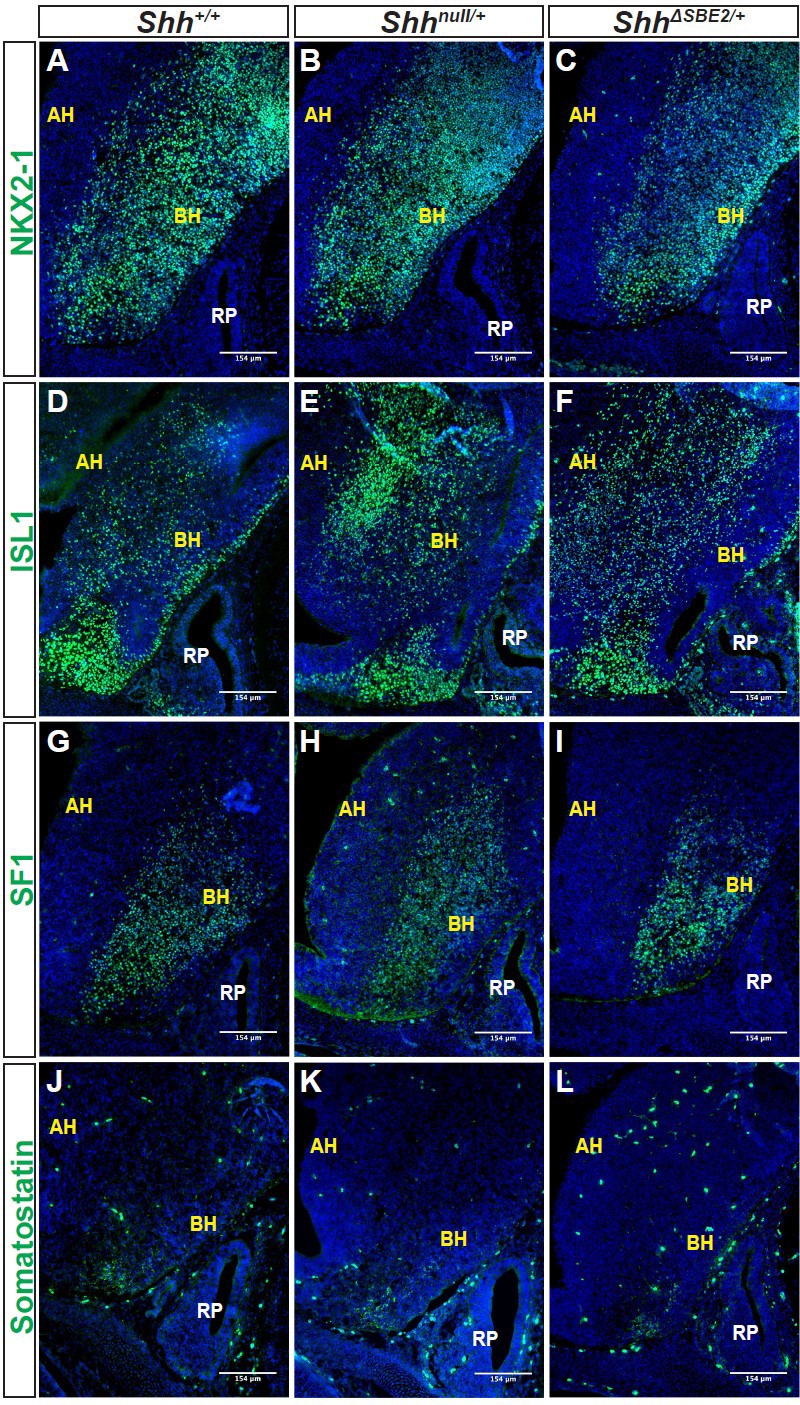


**Supplementary Figure 14. DV hypothalamic neuronal specification is unaffected by heterozygous removal of SBE2 or *Shh* activity.** (A-C) Immunofluorescent staining for NKX2-1 in E13.5 day VH parasagittal cryosections of *Shh+/+*, *Shhnull/+* and *ShhΔSBE2/+* embryos. (D-F) Immunofluorescent staining for ISL1 in E13.5 day VH parasagittal cryosections of *Shh+/+*, *Shhnull/+* and *ShhΔSBE2/+* embryos. (G-I) Immunofluorescent staining for SF1 in E13.5 day VH parasagittal cryosections of *Shh+/+*, *Shhnull/+* and *ShhΔSBE2/+* embryos. (J-L) Immunofluorescent staining for somatostatin in E13.5 day VH parasagittal cryosections of *Shh+/+*, *Shhnull/+* and *ShhΔSBE2/+* embryos. AH- alar hypothalamus; BH- basal hypothalamus; RP- Rathke’s pouch. Scale bars are shown in the bottom right hand corner. (n=3)
